# Effects of a progressive resistance exercise program with high-speed component on the physical function of older women with sarcopenic obesity: a randomized controlled trial

**DOI:** 10.1590/bjpt-rbf.2014.0174

**Published:** 2016-07-11

**Authors:** Karina S. S. Vasconcelos, João M. D. Dias, Marília C. Araújo, Ana C. Pinheiro, Bruno S. Moreira, Rosângela C. Dias

**Affiliations:** 1 Departamento de Fisioterapia, Escola de Educação Física, Fisioterapia e Terapia Ocupacional, Universidade Federal de Minas Gerais (UFMG), Belo Horizonte, MG, Brazil

**Keywords:** physical therapy, obesity, sarcopenia, resistance training, aging, mobility

## Abstract

**Background:**

Sarcopenic obesity is associated with disability in older people, especially in women. Resistance exercises are recommended for this population, but their efficacy is not clear.

**Objective:**

To evaluate the effects of a progressive resistance exercise program with high-speed component on the physical function of older women with sarcopenic obesity.

**Method:**

Twenty-eight women 65 to 80 years old, with a body mass index ≥30kg/m^2^ and handgrip strength ≤21kg were randomly allocated to two groups. The experimental group underwent a 10-week resistance exercise program designed to improve strength, power, and endurance of lower-limb muscles, with open chain and closed chain exercises. The control group had their health status monitored through telephone calls. The primary outcomes were lower limb muscle performance measured by knee extensor strength, power and fatigue by isokinetic dynamometry, and mobility measured by the Short Physical Performance Battery and by gait velocity. The secondary outcome was health-related quality of life assessed by the SF-36 Questionnaire.

**Results:**

The average rate of adherence was 85%, with few mild adverse effects. There were no significant between-group differences for any of the outcomes.

**Conclusion:**

In this study, a progressive resistance exercise program with high-speed component was not effective for improving the physical function of older women with sarcopenic obesity.

## BULLET POINTS

- Sarcopenic obesity (SO) is a disabling condition among older women.
- Resistance exercises are recommended for older people, but its effects for SO are not known.
- We proposed a progressive resistance exercise program with high-speed component for SO.
- This program was not effective for improving the physical function of older women with SO.

## Introduction

In clinical practice, physical therapists are often faced with challenging cases of elderly patients with excess body weight, a condition that has been associated with impairments in muscle performance and functional limitations. Finding the best intervention for these patients can be even more challenging.

The combination of excess body fat and reduced muscle mass or strength is called sarcopenic obesity (SO)^1^ ^,^ ^2^. Older people are at greater risk of SO due to physiological changes during aging. The hormonal decline associated with aging leads to muscle fiber atrophy and accumulation of abdominal and intra-muscular fat, predisposing the patient to SO^1^. A pro-inflammatory state is intrinsically related to this condition and other factors can exacerbate this process such as physical inactivity, comorbidities, and dietary deficiencies^3^. This condition is highly associated with mobility limitations and disability, especially among women^4^ ^-^ ^6^.

The benefits of resistance training for older people are widely recognized, including improvements in muscle performance, functional activities, and quality of life^7^ ^,^ ^8^. Resistance exercises include a variety of techniques and methods aimed at improving muscle strength, power, or endurance. A meta-analysis study concluded that power training with high-speed resistance exercises might be more beneficial to older people than traditional strength training^9^. Resistance exercises have also been recommended as a non-pharmacological therapy for older people with SO to minimize sarcopenia and ameliorate the pro-inflammatory state associated with obesity^10^. Nonetheless, the potential effects of resistance exercises on the physical function of older people with SO are poorly documented. Most studies focus on older people with obesity and frailty or comorbidities and not with the specific condition of SO. Some of them use a combination of different exercises in multicomponent programs and the specific effects of resistance training on physical function cannot be evaluated^10^. In addition, it is not clear what method of resistance exercise is the most suitable or beneficial for this population. Only one study found in the literature has examined the effects of resistance training on the physical function of older adults with SO, comparing high-speed circuit training to hypertrophy training^11^. The authors concluded that the benefits for muscle performance and physical function favored the high-speed circuit as a treatment for SO. However, the exercises for that study were conducted in pneumatic machines, which are not usually available in clinical practice.

The aim of this study was to examine the effects of resistance exercises on the physical function of older people with SO. Therefore, we proposed a resistance exercise program for lower limb muscles, specifically tailored for older women with SO and feasible in clinical practice. The specific research question for this study was “Does a progressive resistance exercise program with high-speed component improve the muscle performance, mobility, and health-related quality of life of older women with sarcopenic obesity?”

## Method

### Experimental design

The Research Ethics Committee of Universidade Federal de Minas Gerais (UFMG), Belo Horizonte, MG, Brazil, approved this study (number ETIC 0172.0.203.000-11). All participants gave written informed consent prior to the data collection.

This study was a prospectively registered, two-arm, randomized controlled trial. The study protocol was described previously as a three-arm trial, with a control group and two intervention groups: land-based and aquatic resistance exercises^12^. However, after the pilot study, the aquatic program was not accomplished due to technical problems with the hydrotherapy pool. Then, the sample size was recalculated for one intervention group of land-based exercises and one control group of no intervention. The other parts of the study protocol remained the same.

The sample size was calculated for a mixed design of repeated-measures analysis of variance to detect between-group differences, estimating an effect size of *f*=0.20 and considering α=0.05, β=0.80, correlation of 0.80 between measures (pre- and post-test) and a possible loss to follow-up of 20%. Therefore, the final sample was calculated as 28 participants, with 14 participants per group.

### Participants

Participants were recruited from databases of previous research projects with community-dwelling older people conducted by our research group on aging. Potential volunteers were contacted by telephone and invited to participate in this clinical trial. In this study, sarcopenic obesity was characterized by a body mass index (BMI) ≥30 kg/m^2^ and handgrip strength ≤21 kg^13^. Women 65 to 80 years old were included in this study. Exclusion criteria were the following: physical, sensory, or cognitive disabilities that could prevent measures assessment or exercise program; cardiovascular, articular, or metabolic diseases in acute or unstable state; concurrent physical therapy treatment for lower limbs; lower-limb fracture or surgery in a one-year period before enrollment in the study.

**Figure 1:**
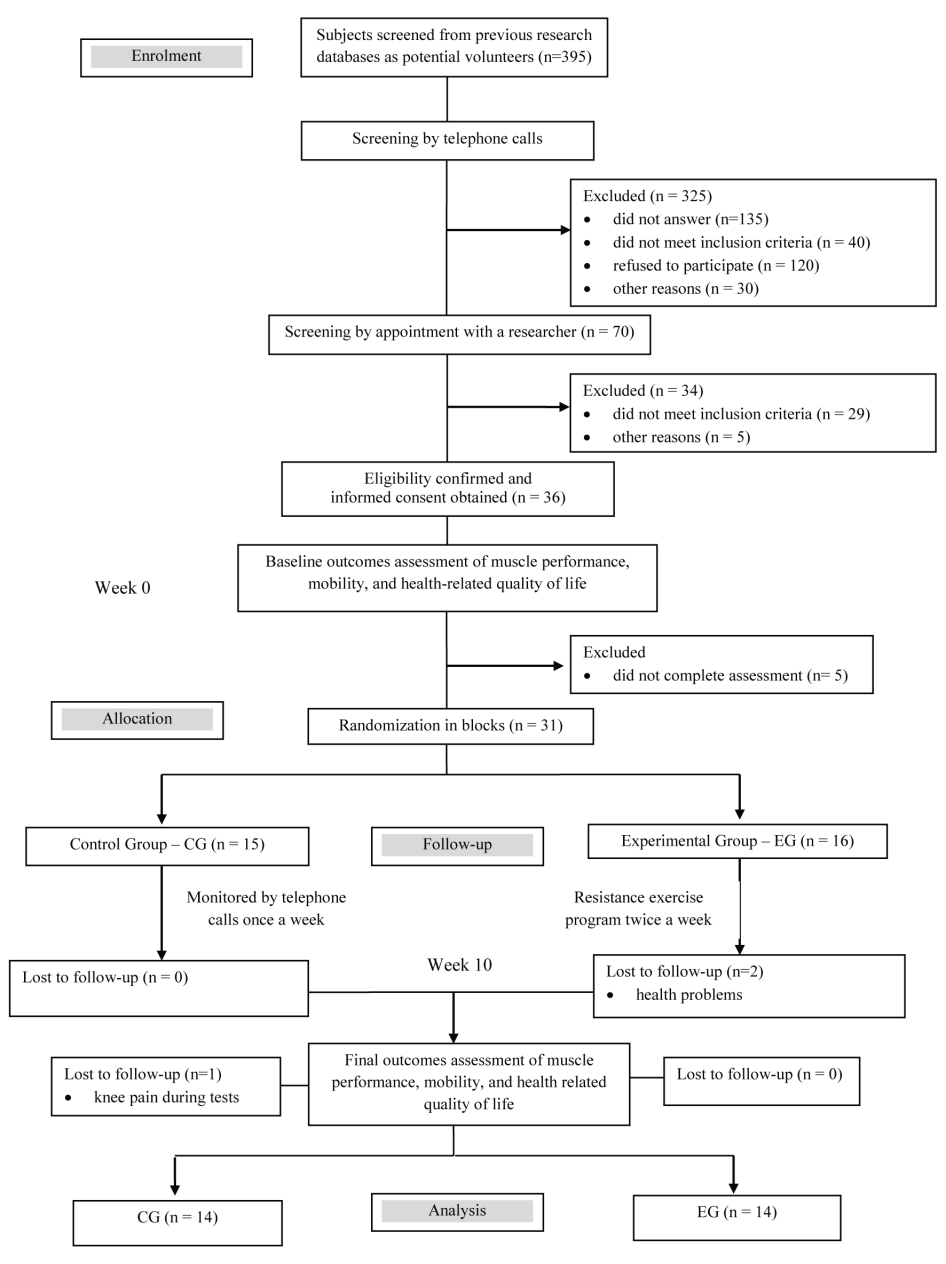
Design and flow of participants in the study.

A total of 395 older women were screened for this study. Thirty-one participants were randomly allocated to one of the groups. The complete flow diagram of participants is presented in Figure 1.

### Procedures

This clinical trial was conducted in the facilities of the Physical Therapy Department of a University. A researcher provided information for eligible women about the procedures of data collection and intervention. Those women who agreed to participate in the trial signed a written consent form.

After signing the informed consent form, a clinical questionnaire was used to collect sociodemographic, anthropometric, and clinical data to characterize the participants. The BMI was calculated as weight per height squared (kg/m^2^). Weight was determined using a calibrated scale (Filizola^TM^, São Paulo, SP, Brazil) and the height was measured with the stadiometer coupled to the scale. The waist circumference (WC) was measured in cm at the umbilicus level with an inextensible fiberglass measuring tape (MABIS®, Briggs Healthcare, Inc., Waukegan, IL, USA). Participants were classified as inactive or physically active accordingly to their score in the Brazilian version of the Human Activity Profile Questionnaire^14^. The Geriatric Depression Scale short form (GDS)-10 was used to evaluate the presence of symptoms of depression in participants (score ≥5)^15^.

Then, participants were evaluated for primary and secondary outcomes. Muscle performance of lower limbs was measured as the knee extensor strength in Joules (J), power in watts (w), and fatigue in percentage (%) using an isokinetic dynamometer (Biodex System 3 Pro; Biodex Medical Systems Inc., Shirley, NY, USA). The strength was calculated as the total work (TW) achieved in a concentric test of five repetitions at 60º/s. Power was calculated as “work/unit of time” during a concentric test of 15 repetitions at 180º/s. The measures of work and power were normalized by body weight. Fatigue index was calculated in a concentric test of 15 repetitions at 180º/s as “(TW_first 5 repetitions_ minus TW_last 5 repetitions_ / TW_first 5 repetitions_) x 100%”. Mobility was measured by the Short Physical Performance Battery (SPPB) in points (0 to 12) and by gait velocity during the 10-Meter Walk Test in m/s. Health-related quality of life was assessed by the Brazilian version of the Medical Outcomes Study Short Form Questionnaire (SF-36) and was represented in this study by the total score of the physical functioning domain.

After a complete baseline assessment, participants were assigned to the control or experimental group according to a computer-generated list of random numbers in block sizes of two. The randomization process was carried out by an external researcher who was not involved in the assessments or interventions. Allocation was concealed from those responsible for providing the exercise program or monitoring the control group until the beginning of the intervention period. These researchers could not be blinded due to the nature of the intervention. After 10 weeks of intervention, the participants underwent another assessment. The outcome assessors were blinded to participant allocation.

Therapists who were not involved in data collection carried out the exercise sessions and monitored the participants in the control group. The therapists of the experimental group were trained to ensure the correct execution of movements during the exercise sessions. The experimental group was monitored for exercise intensity using the modified Borg Scale. The rate of perceived exertion remained between moderate and somewhat severe. The therapists of the control group were trained to interview the participants regarding their health status over the phone.

The experimental group underwent a 10-week resistance exercise program, with 1-hour sessions twice a week. The program was designed to improve lower limb strength, power, and endurance, with open chain and closed kinetic chain exercises. The exercise sessions were performed in groups of up to six participants, accompanied by at least two researchers. Frequency of attendance and occurrence of adverse effects were recorded daily in a clinical form. Each session consisted of a 5-minute walk for warm-up followed by stretching exercises and finally resistance exercises. Stretching exercises remained the same during the entire intervention period. They were performed for 60 seconds in each leg for posterior, anterior, lateral, and medial muscles of hips and knees. In the first 4 weeks of intervention, the resistance exercise program emphasized muscle strengthening and endurance, with concentric and eccentric movements performed at a low speed. Then, the high-speed component was introduced in the program, focusing on muscle power. From the fifth week, the participants were instructed to perform the concentric movements of exercises “as fast as possible”. From the seventh to tenth week, both concentric and eccentric movements were performed at high speeds. The participants were tested in a one-repetition maximum test (1 RM) of knee extensors and flexors before the first session and four weeks later. The complete exercise program is presented at Table 1.

**Table 1:** Description and progression of exercise program.

**Period**		**Exercise**		
Weeks 1 to 10		Stretching exercises - 1 set of 60 s, resting time of 60 s between each exercise		
		Open chain		Closed chain
		In supine position: • Straight leg raise for posterior leg muscles; • Straight leg raise with abduction for medial hip muscles; • Straight leg raise with adduction for lateral hip muscles; • Crossing legs for hip rotator muscles. In prone position: • Knee flexion for anterior leg muscles.		In standing position: • Posterior calf muscles; • Anterior thigh muscles.
Period and loading		Resistance exercises – resting time of 30 s between sets and 60 s between exercises		
Weeks 1 to 2 Concentric and eccentric movements at low speeds		Open chain		Closed chain
• 2 x 12 (50% of 1RM) for knee exercises • 2 x 8 (1 kg) for hip exercises • 2 x 10 for mini-squats, against the wall		In supine position: • Hip flexion with straight leg raise. In lateral positions: • Hip adduction; • Hip abduction.	In prone position: • Hip extension; • Knee flexion. In sitting position: • Knee extension.	In upright position: • Mini-squats with hips in neutral position; • Mini-squats with hips in external rotation.
Weeks 3 to 4 Concentric and eccentric movements at low speeds				
• 2 x 12 (75% of 1RM) for knee exercises • 2 x 8 (2 kg) for hip exercises • 2 x 10 for mini-squats, resting hands on an examination table				
Weeks 5 to 6 Concentric movements at high speeds		Open chain		Closed chain
• 2 x 12 (40% of new 1RM) for knee exercises • 2 x 8 (2 kg) for hip exercises • 2 x 10 for mini-squats (one set at high speed), resting hands on an examination table		In supine position: • Hip adduction. In prone position: • Hip abduction; • Knee flexion. In upright position: • Hip flexion; • Hip extension. In sitting position: • Knee extension.		In upright position: • Mini-squats with hips in neutral position; • Mini-squats with hips in external rotation.
**Weeks 7 to 8** Concentric and eccentric movements at high speeds				
• 2 x 12 (60% of new 1RM) for knee exercises • 2 x 8 (3 kg) for hip exercises • 2 x 10 for mini-squats at high speed, without external support				
**Weeks 9 to 10** Concentric and eccentric movements at high speeds				
• 3 x 12 (60% of new 1RM) for knee exercises • 3 x 8 (3 kg) for hip exercises • 3 x 10 for mini-squats at high speed, without external support				

The control group was monitored by therapists once a week by phone for a 10-week period. This follow-up was designed to ensure that participants in the control group did not engage in lower limb resistance training during this period and to screen for complications that could lead to exclusion from the study. Any information regarding health problems, lower limb pain, medical complications, and medication changes was recorded on a clinical form.

### Statistical analysis

The baseline characteristics of the sample were described in frequencies for categorical variables and measures of central tendency and dispersion for continuous variables. The outcomes data presented normal distribution, according to the Kolmogorov-Smirnov test (P>0.05). The effects of intervention were evaluated by intention-to-treat analysis, including all of the participants who completed the final assessment of outcome measures according to the group to which they were randomly allocated. The within- and between-group differences were calculated for each outcome using two-way, mixed-model, repeated measures ANOVA (group and time) with polynomial contrasts and P≤0.05. The Statistical Package for the Social Sciences version 18.0 (SPSS Inc., Chicago, IL, USA) was used for all data analyses.

## Results

The demographic, anthropometric, and clinical characteristics of participants at baseline are presented in Table 2. The groups were similar at baseline.

**Table 2:** Baseline characteristics of participants (n=28).

**Characteristics - mean**±**SD**	**Exercise Group (n=14)**	**Control Group (n=14)**
**Age (yr)**	72±4.6	72±3.6
**Schooling (yr)**	6.8±3.6	6.5±3.8
**Weight (kg)**	73±6.5	76±6.7
**Height (m)**	1.5±0.04	1.5±0.05
**Body mass index (kg/m^2^)**	32±2.3	33±2.9
**Waist circumference (cm)**	104±8.2	103±6.7
**Comorbidities (number)**	5.6±1.9	4.4±1.8
**Regular medicine (number)**	4.0±1.8	5.9±3.2
**Characteristics – n (%)**		
**Physically active**	11 (78)	9 (64)
**Symptoms of depression**	3 (21)	2 (14)

During the ten weeks of intervention, there was one loss to follow-up in the control group and two in the experimental group. The one from control group did not complete the final assessment because of knee pain from osteoarthritis. The two from the experimental group had health problems not related to the resistance exercise program, i.e. unstable arterial hypertension and an episode of fall at home (Figure 1).

Adherence to the resistance exercise program was high, with an average rate of 85% in the 20 sessions. Low back pain, musculoskeletal pain, or cramps in the lower limbs were the most common adverse effects. In general, these adverse effects were ameliorated with postural corrections or with physiological adaptations through the intervention period. Four participants reported the use of analgesics after one of the first sessions of the program. None of the participants missed exercise sessions because of these adverse effects.

There was no significant between-group difference in anthropometric measurements after the intervention period. Outcome measures at baseline and follow-up for each group, as well as within- and between-group results are presented in Table 3. There were no significant between-group differences for any of the primary outcomes. For the primary outcome muscle performance, there was only a significant within-group difference for knee muscle power at 10 weeks. Participants in the experimental group improved their knee extensor muscle power by 15 w/kg on average (95% CI 7.5 to 24, P=0.01) in relation to baseline values. For the secondary outcome quality of life, there were no significant within- or between-group differences at 10 weeks.

**Table 3:** Mean±SD of pre- and post-treatment outcomes of exercise and control groups, mean±SD of within-group differences, and mean (95%CI) of between-group differences for primary and secondary outcomes.

**Outcome**	**Exercise Group (n=14)**		**Control Group (n=14)**		**Within-group difference**		**Between-group difference**
	**PRE**	**POST**	**PRE**	**POST**	**Exercise Group**	**Control Group**	Interaction group*time
**Strength (J/kg)**	103.38±7.13	105.74±6.63	104.47±6.74	95.77±8.25	2.36±12.0	-8.70±20.0	-6 (-0.90 to 12)
**Power (w/kg)**	87.55±5.25	103.42±6.84	89.31±5.88	91.38±7.15	15.87±13.8*	2.07±23.8	13 (-1.4 to 28)
**Fatigue (%)**	21.16±3.83	27.56±2.94	24.65±3.06	23.69±3.46	6.4±20.0	-0.96±9.0	7.6 (-4.5 to 24)
**SPPB (points)**	11.00±1.4	11.40±1.0	10.00±1.1	10.57±1.2	0.40±1.3	0.57±1.0	-0.14 (-1.04 to 0.76)
**Gait velocity (m/s)**	1.09±0.10	1.11±0.16	1.04±0.19	1.09±0.11	0.02±0.01	0.05±0.17	0.09 (0.07 to -0.15)
**Physical functioning SF-36 (0 to 100)**	81±21	80±23	72±23	78±22	-1.0±12.4	6.0±13.7	-7 (-11 to 3)

PRE: Pre-treatment; POST: Post-treatment; SPPB: Short Physical Performance Battery.

* P-value<0.05.

## Discussion

This is the first randomized trial to test the effects of resistance exercises for older people with sarcopenic obesity comparing an intervention group to a control group with no intervention. In this study, a progressive resistance exercise program with high-speed component was not effective for improving the physical function of older women with sarcopenic obesity.

In older people, physiological responses to anabolic stimulus can be impaired for a number of reasons. First, the mediating mechanisms between exercise and gains in muscle function are usually diminished or delayed in older people. Typical changes in the endocrine, immunologic, and metabolic systems due to age lead to dysfunctional or maladaptive responses to exercise stimulus^16^ ^,^ ^17^. Furthermore, the pro-inflammatory state that accompanies aging is exacerbated by obesity and disrupts the functioning of both muscle and adipose cells^3^ ^,^ ^18^ ^,^ ^19^. In individuals with SO, the combination of low lean mass and high intramuscular fat also contributes to the reduction in the quality and efficiency of muscle contractions^1^ ^,^ ^20^. Therefore, a higher volume of training may be necessary to observe the same effects that occur in other populations of older women^7^ ^,^ ^21^. All of these factors can make it more difficult to improve the muscle performance of older women with SO after resistance exercise training.

Muscle power is an important determinant of functional capacity in older people, as weakness and slowness of movements are associated with aging^22^ ^,^ ^23^. However, the clinical significance of power training for this population remains unclear. A systematic review concluded that this kind of resistance exercise slightly favors functional outcomes in older people compared to conventional strength training. Nonetheless, findings are conflicting and the studies generally present low effect sizes with large CIs. The authors also reported inconclusive results about the adverse effects and safety of power training for older people^9^.

In this study, we aimed to develop an exercise program that would be feasible in clinical practice. To achieve this, we used common materials and a clinical setting that can be easily reproduced. The duration, frequency, and progression of intensity during the program followed the recommendations of the American College of Sports Medicine for older adults^24^. The low frequency of adverse effects in this study suggests that a progressive resistance exercise program with high-speed component is safe for older women with SO.

Gait velocity is a clinical marker of functional capacity in older people. Impairments in this outcome are associated with disability, falls, and mortality^25^ ^,^ ^26^. In this study, there were no significant within- or between-groups differences in mobility outcomes. The functional characteristics of our sample may explain this lack of effects. In this study, most of the participants were physically active, with high levels of functional performance in mobility tests. A ceiling effect may have prevented significant improvements in these outcomes. Furthermore, functional outcomes are usually difficult to detect in clinical trials with older people due to the interaction of multiple health conditions^23^. Two systematic reviews also observed that resistance exercises have lower effects on physical function and quality of life than on muscle performance^8^ ^,^ ^27^. The same ceiling effect may have influenced the quality of life assessment with the SF-36 in our sample. In addition, the duration of intervention proposed in this study may have been insufficient to observe significant changes in these outcomes.

Our exercise program was designed to focus on muscle performance and not on body composition, thus muscle hypertrophy and fat loss were not expected. Although there was no direct measure of muscle or fat mass in our study, we observed that the BMI and WC of participants did not alter significantly after the intervention period. Balachandran et al.^11^ also did not find any effects of two 15-week resistance exercise programs on the body composition of older women with SO^11^. It is possible that exercise programs associated with lifestyle interventions have more beneficial effects on physical function of older people with SO. Evidence in the literature indicates that a combination of diet-induced weight loss and physical exercise produces the best results for body composition in older people with obesity or SO^10^ ^,^ ^28^. This combination helps to preserve muscle mass during the process of weight loss and to improve physical function. However, most of studies used multimodal programs, including aerobic, resistance, and balance exercises. Therefore, the isolated effects of resistance exercise on older people with SO remain unclear.

Despite a robust study design, this clinical trial has some limitations. Blindness of participants and therapists was not possible in this kind of intervention. The long-term effects of the exercise program were not investigated. Our findings relate only to community-dwelling older women between 65 and 80 years of age. Considering differences in body composition and physical function, the effects of a resistance exercise program might be different in men or in the oldest old. Further studies are needed to explore the most effective training parameters of resistance exercise for older people with sarcopenic obesity. Future research should concentrate efforts to identify the interactions between training parameters and individual characteristics that will provide the best results of resistance exercises for older people with sarcopenic obesity. In addition, it is possible that studies with larger samples would be more effective to detect small effect sizes of this kind of intervention.

## Conclusion

The progressive resistance exercise program with high-speed component proposed in this study was not effective for improving the physical function of older women with sarcopenic obesity. There is still a lack of scientific evidence for resistance exercises in this population. The best training parameters for this intervention also remain controversial.
